# Development of myocarditis and pericarditis after COVID‐19 vaccination: A deeper insight

**DOI:** 10.1002/clc.23996

**Published:** 2023-02-20

**Authors:** Maurish Fatima, Muhammad Aemaz Ur Rehman, Muhammad W. Murad, Huzaifa A. Cheema, Sarya Swed

**Affiliations:** ^1^ Department of Medicine King Edward Medical University Lahore Pakistan; ^2^ Department of Medicine Shanxi Medical University Jin Zhong City China; ^3^ Faculty of Medicine Aleppo University Aleppo Syria

To the Editor,

We have read the letter to the editor by Kleebayoon and colleagues with keen interest and would like to appreciate their comments and opinions concerning our systematic review on the development of myocarditis and pericarditis after COVID‐19 vaccination in children and adolescents.^1^ We have responded on a point‐by‐point basis, with an emphasis on the statement regarding the “plausible diagnosis,” thus indicating why it is necessary to reread the discussion of our manuscript to understand the drawn conclusion.

First, regarding the conclusion of our systematic review, a pooled incidence of myocarditis and pericarditis reported by observational studies was (0.00063%) and (0.000074%), respectively.^1^ Appropriate clinical, lab, ECG (Electrocardiogram), and CMR (Cardiac Magnetic Resonance) criteria were used to diagnose myocarditis and pericarditis, along with a significant history of vaccine administration. In the presence of a temporal association between the vaccine and the development of myocarditis and pericarditis, the most appropriate approach is to consider vaccine‐associated myocarditis and pericarditis as a differential diagnosis. Additionally, myocarditis and pericarditis have previously been reported as a side effect of the smallpox vaccine.^2^ However, the lack of evidence from experimental studies imposes a limitation on the deduction of any cause‐effect association.

Second, Kleebayoon and colleagues have commented that it is challenging to identify a precise clinical relationship due to the minimal information available regarding this condition. Given the data at hand, there does appear to be a temporal relationship between vaccination and the development of myocarditis and pericarditis, and the incidence is higher in younger males.^3^ However, a cause‐and‐effect relationship cannot be established because the underlying mechanism is poorly understood, and no experimental trials support this. These points have been well addressed in the discussion section of the manuscript as well.^1^

Third, according to PRISMA (Preferred Reporting Items of Systematic Review and Meta‐analyses) guidelines, the goal of a systematic review is to collate and synthesize findings from the studies to address a research question.^4^ The manuscript aimed to explore the incidence, clinical presentation, management, and any association of myocarditis and pericarditis with the COVID‐19 vaccines in children and adolescents by incorporating the findings from case reports, case series, and observational studies. We have addressed the research question in our manuscript in light of the information available from the included studies and have highlighted the lack of access to individual patient data among the limitations as well. The influence of confounding variables like the patient's genetic make‐up and the presence of comorbidities on the outcome of a condition and the need to conduct a confirmatory test is the burden of experimental studies, not a systematic review. Last, we are grateful to the colleagues for their insightful comments regarding our review and would like to humbly invite them to reread the published manuscript as all of the comments have already been well addressed in the review article.
